# Effect of Macro-, Micro- and Nano-Calcium Carbonate on Properties of Cementitious Composites—A Review

**DOI:** 10.3390/ma12050781

**Published:** 2019-03-07

**Authors:** Mingli Cao, Xing Ming, Kaiyu He, Li Li, Shirley Shen

**Affiliations:** 1 School of Civil Engineering, Dalian University of Technology, Dalian 116024, China; 13774034082@mail.dlut.edu.cn (X.M.); lili_henan@mail.dlut.edu.cn (L.L.); 2 Henan Institute of Metrology, No. 21, Huayuan Road, Zhengzhou 450008, China; hkyzzhn@163.com; 3 CSIRO Manufacturing, Gate 5, Normanby Road, Clayton VIC 3168, Australia; shirley.shen@csiro.au

**Keywords:** calcium carbonate, hydration process, workability, mechanical properties, durability

## Abstract

Calcium carbonate is wildly used in cementitious composites at different scales and can affect the properties of cementitious composites through physical effects (such as the filler effect, dilution effect and nucleation effect) and chemical effects. The effects of macro (>1 mm)-, micro (1 μm–1 mm)- and nano (<1 μm)-sizes of calcium carbonate on the hydration process, workability, mechanical properties and durability are reviewed. Macro-calcium carbonate mainly acts as an inert filler and can be involved in building the skeletons of hardened cementitious composites to provide part of the strength. Micro-calcium carbonate not only fills the voids between cement grains, but also accelerates the hydration process and affects the workability, mechanical properties and durability through the dilution, nucleation and even chemical effects. Nano-calcium carbonate also has both physical and chemical effects on the properties of cementitious composites, and these effects behave even more effectively than those of micro-calcium carbonate. However, agglomeration of nano-calcium carbonate reduces its enhancement effects remarkably.

## 1. Introduction

Concrete is a kind of multi-component and multi-scale composite. Because of its relatively low price, diverse sources and good durability, concrete is widely used in many kinds of buildings and structures. However, with the massive use of concrete, environmental pollution and resource consumption inevitably happen. Cement is a necessary raw material for concrete production, on the other hand, cement manufacture is also one of the most energy intensive industries among mineral process industries [1]. According to data from the U.S. Geological Survey in 2017, global cement production reached approximately 4.2 billion tons [2] and is expected to increase year by year. Moreover, major growth will be foreseen in some developing countries such as China and India [3]. Thus, resource and energy consumption will be an even more serious problem with the increase in cement production. At the same time, 0.87 tons of carbon dioxide will be generated at per ton of cement produced [2]. So, the large amount of carbon dioxide emissions is another severe problem that needs to be solved. Aggregates are also an important constitution of concrete and aggregate consumption is detrimental to the environment as well.

Due to these reasons, supplementary cementitious materials (SCMs) and mineral admixtures are used in concrete or cement manufacture to substitute partial cement or aggregates. Incorporation of SCMs and mineral admixtures such as fly ash, slag, silica fume and limestone is not only an effective way to reduce the carbon dioxide emissions and sources consumption, but also an economic and environmentally friendly way to produce cement or concrete, because most SCMs and mineral admixtures are industrial waste products [4]. Among these SCMs and mineral admixtures, limestone is widely used as a kind of filler material [5,6,7,8,9], aggregate [10,11,12,13], micro-fiber [14,15,16,17,18,19,20] and early strength agent [21] because of its various scales (macro-, micro- and nano-scale) [2,22], morphologies (bulk, granular and fibrous shape), crystal systems (calcite, aragonite, vaterite and amorphous calcium carbonate) [2].

Limestone can be formed of various minerals such as calcite, aragonite, vaterite and amorphous calcium carbonate [2]. Among these, calcite is the most common and stable. So most natural limestone is formed of calcite [23]. It has been confirmed that incorporation of calcium carbonate will not be detrimental to mechanical properties and even has a positive synergic effect on early-age strength, the hydration process, durability and microstructure of cementitious composites [2,22,24,25,26]. Hence, much research has been conducted to clarify the effect mechanism of calcium carbonate on cement paste, mortar or concrete [7,9,27]. In 1938, Bessey et al. [2] first found the formation of calcium-carboaluminate in the hydration process of cement when calcium carbonate was incorporated, which was called the chemical effect of calcium carbonate, and similar results have been found in later studies [2,22,28,29,30,31,32,33]. Subsequently, a large number of studies were conducted on the role of calcium carbonate in cement paste, mortar or concrete. Now it is widely accepted that the density of the matrix can be increased when calcium carbonate is incorporated, because of its filler effect, and the hydration process can be accelerated because of its nucleation effect [2,22,34]. When the particle size of calcium carbonate is comparable to cement grains, the dilution effect will be effective to influence the workability and hydration process of cement [2,35,36]. However, these effects are not independent and often have a coupling effect on mechanical properties, the hydration process, workability and durability of cementitious composites because of its particle size, content and morphology.

**Figure 1:**
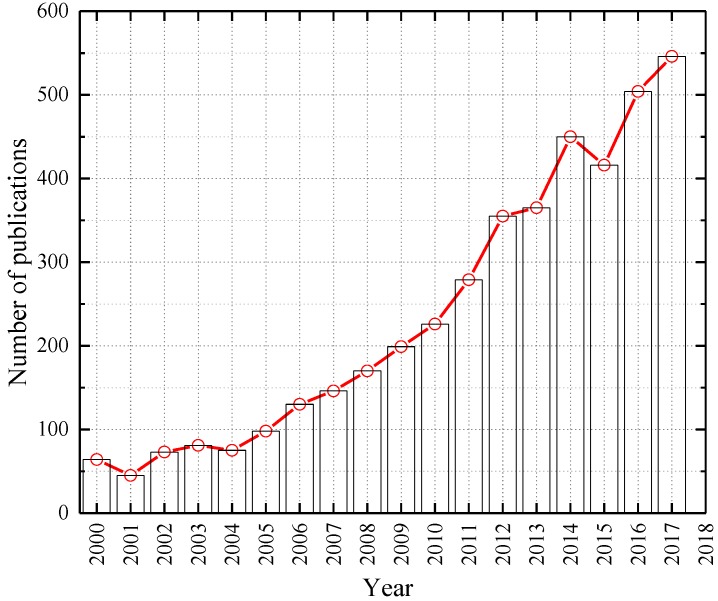
Number of publications per year indexed in Web of Science matching keywords of “concrete and limestone” in search of “Topic”.

Figure 1 shows the number of publications about using limestone in concrete from 2000 to 2017. Applications of limestone is still a hotspot attracting many researchers, especially in recent years. Based on extensive references and research on the effects of calcium carbonate on properties of cementitious composites, many standards have been set to guide the use of calcium carbonate (limestone) in interground and blended cement production, and these standards are sorted chronologically in Table 1. It can be found that calcium carbonate is widely used in many countries acting as aggregates, fillers or admixtures and its content varies from country to country because of its various applications and particle sizes.

Because many studies have been conducted on the effects of calcium carbonate on properties of cementitious composites from fresh mixtures to hardened products, this review focuses on particle size of calcium carbonate and the influence of macro-, micro- and nano-calcium carbonate on the hydration process, mechanical properties, workability and durability of cementitious composites. Moreover, through the summaries of previous references, some constructive suggestions and expectations are proposed as well.

## 2. Macro-Calcium Carbonate

Macro-calcium carbonate refers to calcium carbonate with particle sizes of a millimetric (>1 mm) level, such as coarse limestone aggregates [10,11] and coarse limestone sand [13]. At this scale, the chemical and nucleation effects of calcium carbonate are not significant and thereby the influence of macro-calcium carbonate on the hydration process is negligible. However, the water absorption, particle size and constitution of macro-calcium carbonate aggregates (coarse limestone aggregates) are effective to influence the workability, mechanical properties and durability of cementitious composites.

### 2.1. Workability

The coarse and fine aggregates generally occupy 70–80% of the concrete volume and the water absorption of coarse aggregates significantly influences the fresh properties of cementitious composites [11]. It has been found through investigation that slump loss of fresh concrete is most significant in the first 15 min and dry coarse limestone aggregates causes a higher slump loss compared with the wet one because fresh concrete containing dry coarse limestone aggregates have a higher effective water to cement ratio [11]. Moreover, the workability of fresh concrete also depends on the surfacing filling and particle size of the coarse limestone aggregates. When the fineness modulus of aggregates decreased, the coarse limestone aggregates ratio decreased. Thus, more water is required to achieve the desired workability [37]. 

### 2.2. Mechanical Properties

The mechanical properties of concrete depend on water absorption, particle size and constitutions of coarse limestone aggregates. Incorporation of wet coarse limestone aggregates can generate a concrete with higher compressive strength compared with incorporation of the dry one [11]. In addition, utilization of smaller particle size aggregates may produce a higher compressive strength, as shown in Table 2 [37]. When the coarse limestone aggregate dimension is 0–5 mm, the compressive strength of hardened concrete is up to 42.12 MPa (w/c = 0.33–0.36, 28 d). At the same time, when partial mountain sand is replaced by limestone aggregates with a grain size less than 5 mm, the drying shrinkage of hardened concrete will also decrease [38]. 

The constitution of coarse limestone aggregates influences the strengths and elastic modulus of concrete, especially for high strength concrete (HSC). Due to its low water to cement ratio, the strengths of HSC are determined by the strengths of aggregates, rather than the bond strength between cement paste and coarse aggregates [39,40]. Therefore, it is the mineralogy and strength that control the ultimate strength of HSC. Compared with the different constitutions of coarse limestone aggregates such as calcareous limestone aggregate (85% calcite), dolomitic limestone aggregate (80% dolomite) and quartzitic-gravel aggregate containing schist, dolomite limestone concrete has the highest compressive strength [40]. Beshr and Almusallam [39,40] also obtained similar results when comparing four kinds of coarse aggregates (calcareous limestone, dolomitic limestone, quartzite limestone and steel slag). In addition, they also found that the steel slag concrete had the highest split tensile strength and elastic modulus, followed by that of concrete specimens prepared with the quartzitic, dolomitic and calcareous limestone aggregates because of soft nature of calcareous limestone aggregates. These results have also been proved by the loss on abrasion in different coarse aggregates as shown in Table 3 [39]. However, incorporation of some SCMs such as silica fume, the split tensile strength may increase because of the reaction of calcium hydroxide (Ca(OH)_2_) and silica fume. Thus, concrete prepared with mineral aggregates, such as dolomitic and calcareous limestone aggregates, has a significant improvement in split tensile strength, especially for 90d-strength [40].

### 2.3. Durability

According to what is known, incorporation of limestone aggregates in concrete can affect its durability [25], especially the acid resistance and fire resistance.

#### 2.3.1. Acid Attack 

Concrete used for sewer structures is often attacked by sulfuric acid converted from hydrogen sulfide by bacterial action [13]. To reduce the damage of concrete in an acid condition, there are two effective ways. First, incorporation of SCMs such as fly ash and silica fume in concrete is effective in the reduction of acid attack because of the decreased presence of Ca(OH)_2_, which reacts with acid [41]. Second, usage of a sacrificial medium can reduce the acid concentration near the concrete surface and decrease the rate of deterioration in concrete subjected to acid attack. Calcareous limestone aggregates could act as a sacrificial medium to neutralize the acidic environment and reduce the pH value [13]. In addition, the calcareous limestone aggregate concrete has an excellent sulfuric acid attack resistance ability when SCMs are incorporated in it.

#### 2.3.2. High Temperature Exposure

The compressive strength of concrete after exposure to high temperatures significantly depends on the type of aggregates. The compressive strengths of limestone and siliceous aggregate concrete after exposure to high temperatures have been compared [42,43]. Limestone aggregate concrete has a higher thermal stability compared with the siliceous aggregate concrete, because quartz in siliceous aggregates polymorphically changes at 570 °C with a volume expiation but the decomposition of calcium carbonate is at 800–900 °C [42,44]. However, due to the functions of internal autoclaving, secondary hydration of unhydrated clinkers and SCMs, and the pozzolanic effect, the post-fire strength of concrete may have an increasing trend before 300 °C [43,44,45], especially for the concrete made with siliceous aggregates [43]. Note that when the temperature exceeds 800 °C, concrete would deteriorate irreversibly regardless of being prepared by limestone or siliceous aggregates.

In conclusion, macro-calcium carbonate such as coarse limestone aggregate plays an important role in controlling workability, mechanical properties and durability of cementitious composites. Incorporation of macro-calcium carbonate in cementitious composites can improve both ambient and post-fire strengths. Moreover, macro-calcium carbonate can be regarded as an inert filler.

## 3. Micro-Calcium Carbonate

Micro-calcium carbonate (1 μm–1 mm), such as limestone powder and limestone dust, is widely used in cement manufacture as a kind of blended or interground material. Though micro-calcium carbonate has no pozzolanic activity and cannot react with alkaline substances such as Ca(OH)_2_ and calcium oxide (CaO), incorporation of micro-calcium carbonate in cement can have both physical and chemical effects on the hydration process, workability of fresh mixture and mechanical properties of hardened products. Thus, it is imprecise to regard micro-calcium carbonate as an inert filler, especially when micro-calcium carbonate has a smaller particle size than cement grains or is incorporated in ternary or quaternary blends containing SCMs such as fly ash and metakaolin; in these situations micro-calcium carbonate may participate in the cement hydration process and affect the factors of hydration kinetics and microstructure [46,47,48]. Finally, the mechanical properties and durability will also be influenced. Therefore, the effect of micro-calcium carbonate on the hydration process, workability, mechanical properties and durability is reviewed in the following section.

### 3.1. Hydration Process

As a micro-calcium carbonate, limestone powder has a higher specific area and surface energy than that of macro-calcium carbonate. So the effects of limestone powder on accumulative hydration heat, the release rate of hydration heat and the hydration products of cementitious composites are different from that of macro-calcium carbonate and mainly affected by various particle size, content and crystal structure of micro-calcium carbonate. Table 4 shows the main action mechanism of limestone powder on the hydration process of cement paste. According to this table, the main action mechanism of limestone powder on the hydration process is discussed through the following aspects of particle size, content and crystal structure. 

#### 3.1.1. Particle Size

**Figure 2:**
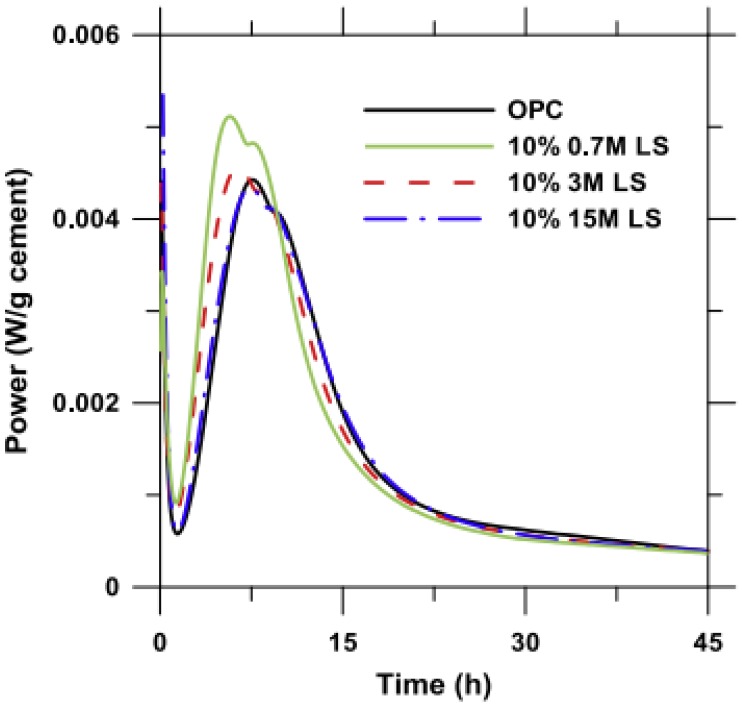
Influence of particle size on the heat release rate [47].

**Figure 3:**
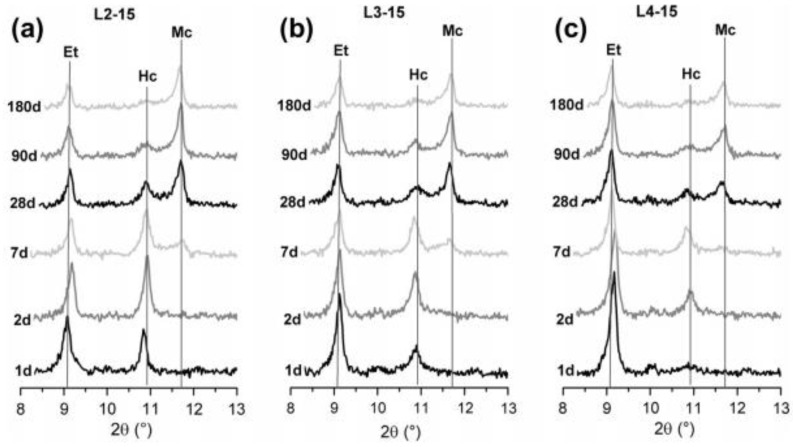
Influence of different quantities of anhydrite on the hydration of laboratory limestone (LS) containing Portland cements: (**a**) 2.1% CaSO_4_ + 15% LS, (**b**) 3% CaSO_4_ + 15% LS, and (**c**) 3.8% CaSO_4_ + 15% LS. The main reflexes of ettringite (Et), hemicarbonate (Hc), monocarbonate (Mc) are indicated [51].

The particle size of micro-calcium carbonate affects its physical effects (the filler effect, dilution effect and nucleation effect) and chemical effects. When a coarser (comparable or coarser than cement grains) limestone powder is used in cementitious composites, the main action effect of limestone is the filler effect. Because of the smaller surface energy and lower dissolution in an alkaline environment, limestone powder hardly participates in the hydration process of cement and may only fill the voids between aggregates such as sand and coarse aggregates. However, when a finer (finer than cement grains) limestone powder is incorporated in cementitious composites, accumulative hydration heat, the release rate of hydration heat and the hydration products are all greatly different. For the chemical effect of micro-calcium carbonate, the results may be interesting. Vance et al. [47] investigated the particle size of limestone powder on cement hydration and three limestone powders of different fineness were used. The finer limestone powder (median particle size = 0.7 or 3 μm) significantly accelerates the hydration process of calcium silicate and increases the hydration peak (see in Figure 2 [47]), because finer limestone powder has a larger specific area and surface energy and provide additional nucleation sites for the formation and development of calcium silicate hydrate (C-S-H) [5,9,26,35,47,50,51], which is known as nucleation effect. Moreover, the second hydration peak is generally recognized as the hydration of calcium aluminate and will demonstrate a significant improvement when 0.7 μm limestone powder is incorporated, which means the formation of new hydration products such as hemicarboaluminate and monocarboaluminate. The formation of carboaluminates has also been confirmed by many other researchers [5,26,30,51]. However, hemicarboaluminate is not thermostable and mainly exists in the early hydration process (before 7 d), and then slowly converts to monocarboaluminate. The formation of carboaluminate depends on many factors such as kinetics of hemi- and monocarboaluminate formation and the dissolution of calcium carbonate is lower in high pH conditions, which causes the actual amount of calcium carbonate participating in the formation of carboaluminate to be far less than the content of limestone powder [51]. Therefore, the intensity of the carboaluminates peaks in the X-ray diffraction (XRD) pattern is lower and difficult to detect compared to other hydrates, as shown in Figure 3 [51]. For the second hydration peak, Bentz et al. had a similar result through the investigation of hydration of cement prepared with different fineness of limestone powder [50], and another possibility for the increasing second hydration peak of cement containing fine limestone powder (nano-limestone powder and 4.4 μm limestone powder in reference [50]) may be that limestone powder can provide an additional source of calcium irons to the pore solution, even though calcium carbonate has a relatively low dissolution in the elevated pH condition [50]. When a coarser limestone powder (15 μm in reference [47]; 20 μm in reference [35] and 15.7 μm in reference [26]) is used in cementitious composites, the dilution effect is also significant. Though the heat release rate of coarse limestone powder-cement is still higher than that of the pure cement, the total hydration heat is comparable or even lower than that of pure cement, as shown in Figure 2 [47].

#### 3.1.2. Content

**Figure 4:**
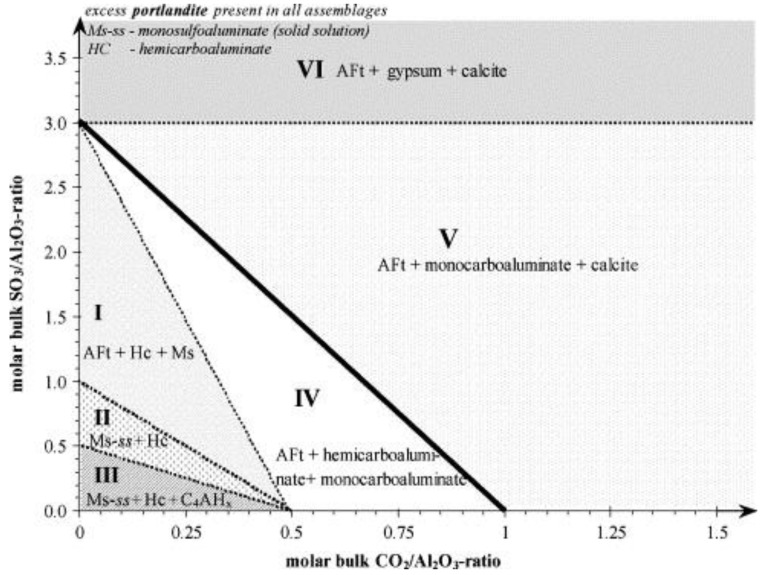
Calculated phase assemblages of a hydrated mixture consisting of C_3_A, Ca(OH)_2_ and varying initial sulfate (SO_3_/Al_2_O_3_) and carbonate ratios (CO_2_/Al_2_O_3_) at 25 °C (molar units) [24].

The content of limestone powder can also affect the main action mechanism of limestone powder on cement hydration. In general, the nucleation effect increases with the increase of limestone powder content. This is because more nucleation sites can be provided for the formation of C-S-H and the accumulative hydration. The heat release rate will also increase. The effect of the content of limestone powder on its chemical effect may be complicated for the following two reasons: (1) the formation of hemi- and monocarboaluminate mainly depends on the kinetics rather than the amount of calcium carbonate present; and (2) the dissolution of calcium carbonate is small and the content of aluminate in cement is low as well [51]. However, some quantitative relationships of the chemical effects of limestone powder can still be calculated according to the chemical reaction equations, and the results are shown in Figure 4 [24]. Regions I, II and III are delineated by dotted lines, which means the hydrates in these areas are metastable phases. According to the boundaries in the three areas, the content of carboaluminates is the function of the content of sulfate, carbonate and aluminate. There is no calcite in regions I to IV, which means calcite totally participates in the reaction of calcium carbonate and tricalcium aluminate (C_3_A). But in regions V and VI, the calcite just acts as an inert filler to fill the voids between cement grains. Conversely, the dilution effect has a significant enhancement with the increase of limestone powder content, especially for the ultra-fine limestone powder [2]. Because more free water can be substituted by the ultra-fine limestone powder in voids, the effective water to cement ratio increased.

#### 3.1.3. Crystal Structure

**Figure 5:**
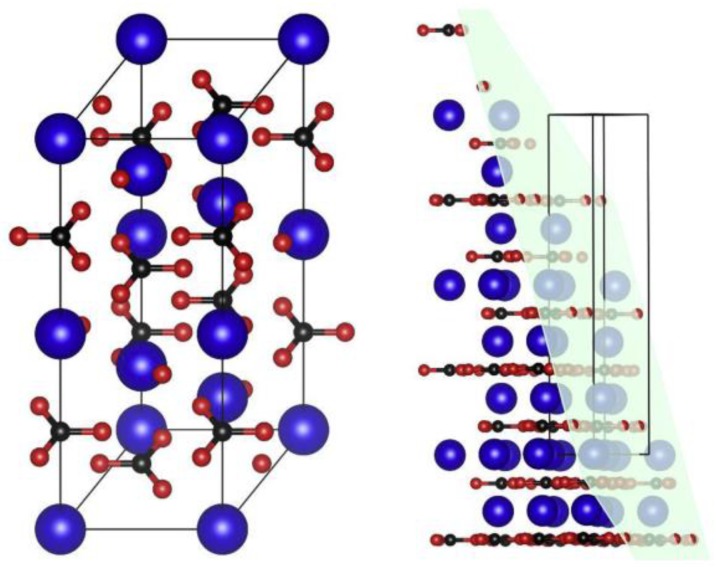
Calcite unit cell general view (**left**) and dissected by (10$\overset{-}{1}$4) plane (**right**). Atom radius is reduced from actual values to allow better visualization. (Ca: large blue atoms; C: small black atoms; O: small red atoms) [52].

Limestone powders with different crystal structures may have different influences on cement hydration. The influence of aragonite (sturcal F) and calcite (heat-treated sturcal F) on cement hydration have been investigated [26]. Calcite can significantly accelerate the hydration process, but aragonite may not. As shown in Figure 5 [52], the planar configuration of calcite consists of Ca and O atoms, which is similar to the CaO layer in C-S-H gel. But to aragonite, only Ca atoms are detected in the surface layer for aragonite. So, calcite has an improvement on hydration process. However, because of the similar dissolution processes of calcite and aragonite in an ambient environment, the chemical effect may not be distinguished [26].

### 3.2. Workability

The workability of a fresh mixture containing limestone powder mainly depends on its particle size, content and surface morphology. The viscosity (tested by V-funnel time or rheometer) increases with the decrease in particle size of limestone powder, especially when the particle size is comparable or smaller than those of cement grains, because of the fill effect and higher specific area of limestone powder [53]. Therefore, self-compacting concrete (SCC) prepared with fine limestone powder (median particle size < 20 μm [53,54,55]) may have a good segregation resistance ability and workability. The viscosity is also influenced by the replacement content of limestone powder, but it is not a linear relationship between the replacement content and variation of viscosity [53]. The effects of limestone powder on yield stress (tested by spread flow or rheometer) may be complicated. Coarse limestone powder (Blaine fineness = 4430 cm^3^/g) could reduce the spread flow values (increased the yield stress), but fine limestone powder (Blaine fineness = 5380 cm^3^/g) increased the spread flow values (decreased the yield stress) [53]. Bentz et al. [52] used a finer limestone powder than that in reference [53] and found that fine limestone powder could increase the flowability and decrease the yield stress. Cao et al. also investigated the effect of morphology of calcium carbonate on viscosity and yield stress of cement mortar containing aragonite calcium carbonate whisker (CW) with a needle-like shape (aspect ratio = 10–60) [56]. Both viscosity and yield stress increase with the increased substitution amount of cement because of its higher specific area. The purity of limestone powder [57] also affects the workability of fresh mixture in addition to the above factors.

### 3.3. Mechanical Properties

#### 3.3.1. Limestone Powder

The mechanical properties of cementitious composites containing limestone powder depend on particle size, content and morphology. With the decrease particle size, compressive strength at early-age (before 7d) is found to increase with a constant content of limestone powder [1,47,54,55]. But for long-term age, incorporation of finer limestone powder may decrease the compressive strength [1], because the dilution effect of finer limestone powder may be more effective than its filler effect or nucleation effect at the end stage of the hydration process. With the increased content, compressive strength and flexural strength decrease [1,47,54,55,58]. On one hand, a high replacement content reduces the amount of cement and this is not good for the strength development because limestone powder has no cementitious ability. On the other hand, the dilution effect is more effective with the increase in substitution content, and causes a high effective water to cement ratio and lower strength. However, flexural defection of polyvinyl alcohol fiber reinforced engineered cementitious composites (PVA-ECC) may be improved after the addition of limestone powder because of uniform dispersion of PVA fiber caused by the diluting effect of limestone powder [58].

#### 3.3.2. Calcium Carbonate Whisker

**Figure 6:**
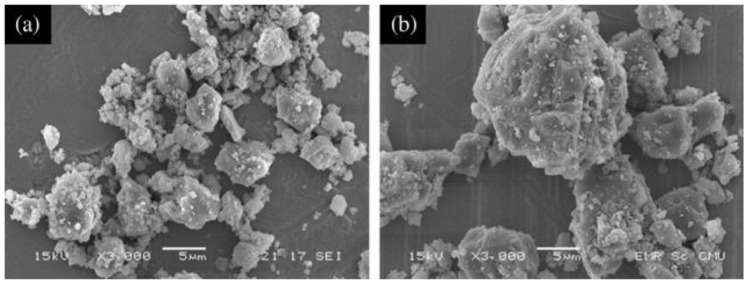
Scanning electron microscopy (SEM) images of limestone powder with particle size of (**a**) 5 μm and (**b**) 20 μm [35].

**Figure 7:**
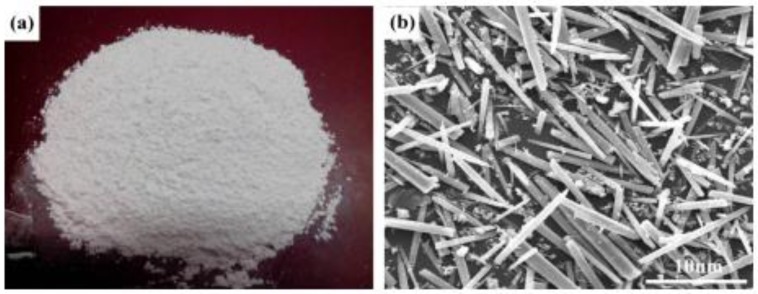
Images of (**a**) macro-morphology of calcium carbonate whisker (CW); (**b**) micro-morphology of CW as shown by SEM [56].

Calcium carbonate whisker (CW) was first used in the paper industry to enhance the toughness of paper. It is different from limestone powder with a bulk shape [35] or granular shape (see in Figure 6), but is needle-like [56] (see in Figure 7). Because of its shape, CW not only fills the voids to make the matrix dense [14] but also plays a role in resisting the development of micro-cracks, especially with incorporated steel fiber [16,20,59], PVA fiber [15] and carbon fiber [60]. Moreover, a positive synergic effect can be demonstrated when incorporated of a hybrid fiber system [61,62].

### 3.4. Durability

#### 3.4.1. Acid Attack 

In some special environments, concrete may be attacked by acid. Because of the reaction between Ca(OH)_2_ produced by the hydration process and acid ions, a high weight loss may occur and, therefore, cause the deterioration of strength. Incorporation of limestone power in cementitious composites can reduce the weight loss [55,63]. Moreover, with the increase of substitution content and decrease of particle size, cement mortar or concrete containing limestone powder exhibit a better resistance to acid attack [55]. This is because less Ca(OH)_2_ is produced when the replacement content of cement is higher. In addition, a finer limestone powder may have a more effective filler effect and make a denser matrix [55]. Thereby, the incorporation of more and finer limestone powder in cementitious composites may give a better resistance to acid attack, to some extent.

#### 3.4.2. High Temperature Exposure

Incorporation of limestone powder in cementitious composites may be not good for their ability to resist high temperature exposure. With the increases of temperature and/or content of limestone powder, there are decreases of the compressive strength, ultrasonic pulse velocity (UPV) decrease and increase of weight loss [64,65], especially after the decomposition of calcium carbonate at around 800–900 °C [42,44].

However, it is noticeable that limestone powder used in these references [64,65] is coarser than cement grains and causes a more effective dilution effect, especially when more cement is replaced. Therefore, the residual is lower compared to that without limestone powder before 600 °C. In addition, there are not enough studies about the effects of limestone powder on the properties of high temperature-damaged cementitious composites, especially when the fineness and crystal structure of limestone powder are taken into consideration. So, more studies are needed in this area.

In conclusion, micro-calcium carbonate can affect the hydration process of cement by its dilution effect, nucleation effect and chemical effect. These effects are significantly influenced by the particle size, content and crystal structure. Workability of fresh mixture is also influenced by the particle size and content through the filler effect. Subsequently, mechanical properties and durability are also influenced because of the effect of micro-calcium carbonate on the hydration process and workability. Moreover, the main difference between macro- and micro-calcium carbonate is that micro-calcium carbonate has a chemical effect on cementitious composites, except for the physical effect (filler effect), especially incorporation of finer micro-calcium carbonate (limestone powder) in cementitious composites.

## 4. Nano-Calcium Carbonate

Nanoparticles are commonly defined as materials with a particle size of less than 100 nm [66,67], and can make revolutionary changes in bulk material properties [68]. Incorporation of nanoparticles in cementitious composites can significantly improve their mechanical properties and durability [67,69,70,71,72]. Among these nanoparticles, nano-calcium carbonate is one of the most widely used nanoparticles in the construction sector. In order to distinguish micro-calcium carbonate and nano-calcium carbonate, the particle size of nano-calcium carbonate is less than 1 μm, rather than being more strictly defined as less than 100 nm. Compared with micro-calcium carbonate, nano-calcium carbonate has a finer particle size and larger specific area, and thereby a more significant effect on the hydration process, workability, mechanical properties and durability of cementitious composites can be observed, even only with a small amount. 

### 4.1. Hydration Process

**Figure 8:**
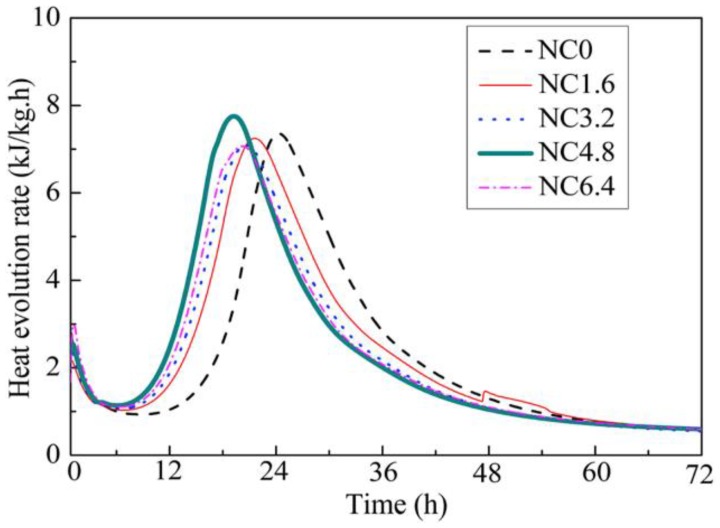
Effects of nano-calcium carbonate contents on heat evolution rate of ultra-high strength concrete (UHSC) [77].

The effect of nano-calcium carbonate on the hydration process of cement depends on its content, particle size and crystal structure [22,73,74,75,76,77,78,79]. Sato et al. [73,79] studied the influence of content and particle size of nano-calcium carbonate on cement hydration. Nano-calcium carbonate (50–120 nm) is very effective in accelerating the cement hydration, especially for the induction period of tricalcium silicate (C_3_S), because of its nucleation effect [79]. Moreover, with the increase of calcium carbonate content, the acceleration effect of nano-calcium carbonate is more and more pronounced and the hydration peak of tricalcium aluminate (C_3_A) and tetracalcium aluminoferrite (C_4_AF) is also more and more remarkable. Similar results are also shown in Figure 8 [77]. Both the dormant period and the appearance of the second hydration peak (associated with hydration of C_3_A and C_4_AF) are shortened. The reasons are that the calcium ions can be absorbed onto the surface of nano-calcium carbonate when the C_3_S is dissolved in water because of the high surface energy of nano-calcium carbonate, and thereby it causes the concentration reduction of calcium ions around the C_3_S. It is favorable for accelerating the reaction of C_3_S. In addition, dissolved carbonate ions from nano-calcium carbonate can react with C_3_A to form hemi- and monocarboaluminates [76,77]. However, nano-calcium carbonate can also react with C_3_S to form C-S-H gel and Ca(OH)_2_ and this may be also the reason for earlier and higher hydration heat. The dilution effect of nano-calcium carbonate can also be found in Figure 8 because the mixture containing 4.8% ( by weight) nano-calcium carbonate (15-105 nm, 97.8% calcite) has a higher and earlier hydration heat [77], which means nano-calcium carbonate is more effective to perform a dilution effect compared with micro-calcium carbonate (micro-calcium carbonate performs a dilution effect when its content is 10% (by weight) discussed in Figure 2 [47]). 

**Figure 9:**
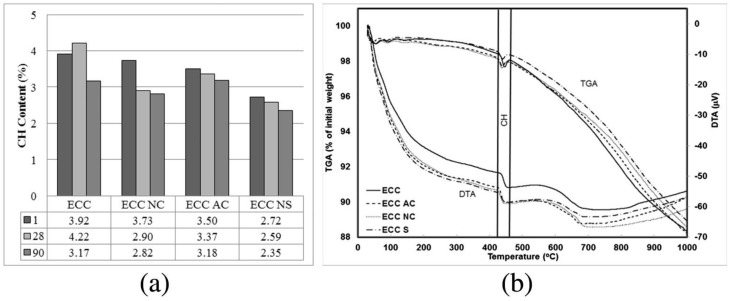
(**a**) Change of Ca(OH)_2_ content of engineered cementitious composite (ECC) mixtures with age. (**b**) Typical series of thermogravimetric analysis (TGA/DTA) curves of mixtures at the age of 28 days [78].

Crystal structure of nano-calcium carbonate can also influence the cement hydration process. The influence of calcite nano-calcium carbonate (NC) and aragonite nano-calcium carbonate (AC) on the properties of PVA-ECC are investigated [78]. From the thermogravimetric analysis (TGA/DTA) in Figure 9, ECC containing AC has a similar Ca(OH)_2_ content compared with the control group at 90d because the surface structure of aragonite calcium carbonate is less favorable for the formation of C-S-H [78], which means AC is less effective to accelerate the hydration process compared with the NC. These results are similar to that for micro-calcium carbonate [26]. However, the Ca(OH)_2_ content decreases with the increase of age because of the formation of carboaluminates, carbonation and pozzolanic effect (only for nano-silicon oxide in this reference).

### 4.2. Workability

The workability of cementitious composites with incorporated nano-calcium carbonate depends on content and particle size. In general, with an increase in particle size or content, the yield stress (spread flow) and viscosity (V-funnel time) increase [27,80,81]. However, when particle size and content of nano-calcium carbonate are taken into consideration at the same time, their combined effect on the workability may be different from the effect of each one. It is generally recognized that demanding water of cementitious composites includes two aspects: (1) filling water in the voids between the cement grains; and (2) absorbing water on the surface of cement particles [77]. In addition, the action mechanism may also include two aspects: (1) the dilution effect, which means water in voids can be substituted by nano-calcium carbonate particles; and (2) the filler effect, which means finer nano-calcium carbonate particles can fill the space between cement particles and at the same time, more free water can be absorbed onto the surface of nano-calcium carbonate because of its larger specific area and higher surface energy. Both the two action mechanisms can be influenced by particle size and content of nano-calcium carbonate, and thereby with the increase of content, the flowability may perform differently [22,75,82].

### 4.3. Mechanical Properties

The mechanical properties of cementitious composites containing nano-calcium carbonate mainly depend on their contents. Flexural strength initially increased up to a nano-calcium carbonate (15–40 nm) addition rate of 2% (by weight) and then decreased [78]. With incorporation of nano-calcium carbonate in PVA-ECC, the mid-span deflection significantly improves, especially at early-age (before 1 d). However, comparing the effect of calcite and aragonite nano-calcium carbonate on flexural and compressive properties, the calcite is more effective because it is more favorable to accelerate the formation of C-S-H [78]. For compressive strength, with the increase in nano-calcium carbonate content, compressive strength initially increases and then decreases [27,77,81,83]. The reasons are that, on one hand, nano-calcium carbonate can accelerate the hydration process and react with C_3_S and C_3_A to form C-S-H and carboaluminates, and this effect is more effective with the increase of content to some extent. However, when a large amount of cement is replaced by nano-calcium carbonate, the dilution effect is more effective, just like the micro-calcium carbonate. Moreover, agglomeration of nano-calcium carbonate will seriously reduce the development of compression, which is different to micro-calcium carbonate [1,54,55,58]. In addition, the denser matrix caused by the addition of nano-calcium carbonate could not provide available space for the formation of hydration products [77]. In general, incorporation of nano-calcium carbonate in cementitious composites can improve early-age strength and incorporation of SCMs may be helpful for long-term strength [84], so the hybrid use of nano-calcium carbonate and SCMs may have a synergic effect on both early-age and long-term strength.

### 4.4. Durability

Very few studies about the effects of acid attack on properties of cementitious composites containing nano-calcium carbonate can be found. However, according to other durability tests such as water sorptivity and chloride permeability [80,85], incorporation of nano-calcium carbonate in cementitious composites can make the matrix dense and reduce the pores. Thus, the impermeability and the acid attack resistance ability may be good, especially when the partial replacement of cement is greater.

For high temperature exposure, incorporation of nano-calcium carbonate in cementitious composites can improve its peak compressive stress, ultimate compressive strain, compressive toughness and flexural properties no matter in ambient environment or in/after high temperature [68,86,87]. But it is ineluctable for the rapid decrease in strength after 800 °C because of the decomposition of calcium carbonate.

In conclusion, just like the micro-calcium carbonate, nano-calcium carbonate can also affect the hydration process, workability, mechanical properties and durability through the filler effect, dilution effect, nucleation effect and chemical effect. All these effects are influenced by the content, particle size and crystal structure of the nano-calcium carbonate. However, the effects of nano-calcium carbonate are more effective than those of micro-calcium carbonate, and it is ineluctable that the agglomeration of nano-calcium carbonate is also more remarkable than that of micro-calcium carbonate because of its higher surface energy and larger specific area.

## 5. Summary and Expectation

### 5.1. Summary

The effects of macro-, micro- and nano-calcium carbonate on the hydration process, workability, mechanical properties and durability of cementitious composites have been reviewed. Based on the discussion above, conclusions can be drawn as follows:

(1) Macro-calcium carbonate mainly acts as an inert filler in cementitious composites. The influence of macro-calcium carbonate on the cement hydration process is insignificant. The workability of fresh mixture depends on water absorption and particle size of macro-calcium carbonate, and concrete prepared with dry macro-calcium carbonate has a higher slump loss. The mechanical properties of concrete depend on water absorption, particle size and the constitutions of macro-calcium carbonate and concrete prepared with wet and fine macro-calcium carbonate may have a higher strength. Comparison of different mineral aggregates, macro-calcium carbonate (coarse limestone) aggregates are less favorable for the improvement of strength, but incorporation of SCMs in concrete can offset this problem. Macro-calcium carbonate is not a good material to resist an acid attack because of its soft nature. But it is good for resisting high temperature exposure compared with siliceous aggregate, because of its higher thermostability.
(2) Micro-calcium carbonate has both a physical effect (filler effect, dilution effect and nucleation effect) and a chemical effect on cementitious composites. The cement hydration process depends on particle size, content and crystal structure of micro-calcium carbonate. In general, the finer the micro-calcium carbonate particles are, and the higher the content is, the more significant the acceleration effect of the hydration process will be. Moreover, calcite is more favorable in accelerating the hydration process than aragonite, because of their different crystal surface structures. The workability of fresh mixture containing micro-calcium carbonate powder mainly depends on its particle size, content and surface morphology. A finer powder and higher content may cause a higher yield stress and viscosity, but at the same time the dilution effect is also more effective. Therefore, the workability may not have a clear and linear relationship with the particle size or content when all of these factors work together. Conversely, the influence of CW on workability is clearer, and both viscosity and yield stress increase with increased substitution amount of cement. The mechanical properties of cementitious composites containing micro-calcium carbonate depend on its particle size, content and surface morphology. In general, the improvement of micro-calcium carbonate is effective on early-age strength because of its acceleration effect of cement hydration. Incorporation of micro-calcium carbonate in cementitious composites can make the matrix denser and thereby the acid attack resistance ability is better compared with that without micro-calcium carbonate. Incorporation of micro-calcium carbonate in cementitious composites may not be good for its ability to resist high temperature exposure.
(3) Nano-calcium carbonate can have physical and chemical effects on cementitious composites, and these effects of nano-calcium carbonate are more effective compared with those of micro-calcium carbonate. However, the agglomeration of nano-calcium carbonate is also more effective. The effect of nano-calcium carbonate on the hydration process of cement depends on its content, particle size and crystal structure. The hydration process can be accelerated through the nucleation effect and chemical effect. But the dilution effect decreases total hydration heat. The workability of cementitious composites with incorporated nano-calcium carbonate depends on their content and particles. In addition, yield stress and viscosity will perform differently because of the combined effect of particle size and content. Incorporation of nano-calcium carbonate in cementitious composites can improve early-age strength when a proper amount is used. Hybrid use of nano-calcium carbonate and SCMs has a synergic effect both on early-age and long-term strengths. The resistance ability to acid attack of cementitious composites containing nano-calcium carbonate is not clear, but nano-calcium carbonate can make the matrix denser. Incorporation of nano-calcium carbonate in cementitious composites is favorable for its high temperature behaviors.

### 5.2. Expectation

There have been many studies about the effect of macro-, micro- and nano-calcium carbonate on properties of cementitious composites. Many effective and significant results and mechanisms have been produced and proposed. But further studies are still needed on:

(1) The high temperature properties of cementitious composites containing calcium carbonate particles. On one hand, the activity and chemical constitutions of calcium carbonate may be different in/after high temperatures. On the other hand, whether incorporation of calcium carbonate is favorable for the high temperature properties of cementitious composites needs more study, especially for aragonite, because a crystal transition will happen at around 450 °C and the influence of crystal transition on the properties of cementitious composites is still not clear.
(2) Hybrid use of multi-scale calcium carbonate. Macro- and micro-calcium carbonate are more widely used compared with nano-calcium carbonate because nano-calcium carbonate has a relatively high price and is difficult to be dispersed uniformly. Hybrid use of multi-scale calcium carbonate may be a useful way to solve these problems, and thereby more research needs to be conducted to investigate the feasibility and effectiveness of this method.

## Figures and Tables

**Table 1:** Standards related to use of limestone in cement production in different areas [1,2,3,23,25].

Date	Area	Standard	Content
1987	Europe	–	Define Portland-LS cement with 15% ± 5% LS.
1991	Brazil	NBR 11578/91	Portland-LS cement can contain from 6% to 10% LS fillers.
1999	Mexico	NMX C-414-0/99	Portland-LS cement can contain from 6% to 35% LS fillers.
2000	Europe	EN 197-1	Define different four types of Portland-LS cement containing 6–20% limestone (types II/A-L and II/A-LL) and 21–35% limestone (types II/B-L and II/B-LL), respectively.
2007	China	GB 175-2007	LS can act as an inactive mixture in cement production.
2007	Argentina	IRAM 50000/07	Portland-LS cement can cantina up to 25% of calcareous materials.
2008	Canada	CSA A3001-08	LS content is below 15% of total binder content.
2010	Canada	CSA A3001-10	Portland-LS cement is defined as GUL.
2012	U.S.	ASTM C595	LS content is up to 15% of total binder content.

Note: LS means limestone; GUL represents general use limestone cement.

**Table 2:** Compressive strength of concrete in different water to cement ratio and curing days (unit: MPa) [37].

Aggregate Dimension (mm)	w/c = 0.33–0.36			w/c = 0.3			w/c = 0.4			w/c = 0.5		
	7 d	14 d	28 d	7 d	14 d	28 d	7 d	14 d	28 d	7 d	14 d	28 d
0–5	11.86	19.92	42.12	12.44	20.51	42.34	11.21	19.82	42.08	7.47	16.69	34.95
0–10	11.48	17.25	35.08	9.99	17.09	36.13	13.03	17.42	33.91	5.13	9.56	19.84
0–20	8.04	17.21	35.23	7.46	17.12	34.78	8.78	17.39	35.73	7.48	11.08	22.35
5–10	8.19	14.87	29.75	7.29	14.82	30.33	9.06	14.95	28.69	5.58	10.61	20.39
10–20	8.63	8.56	18.36	4.99	7.27	17.41	6.33	12.31	20.43	3.24	6.46	12.42

**Table 3:** Loss on abrasion in the coarse aggregates [39].

Type of Aggregate	Loss on Abrasion (%)
Calcareous limestone	34.4
Dolomitic limestone	24.2
Quartzitic limestone	19.2
Steel slag	11.6

**Table 4:** Effect of micro-calcium carbonate on cement hydration.

Author (Date) [Reference]	Binder		Particle Size (μm)		Blaine Fineness (cm^2^/g)		Main Action Mechanism
	Mass Content (wt.%)	Volume Content (vol. %)	LS	PC	LS	PC	
Bonavetti et al. (2001) [30]	(20%) LS + (80%) PC	–	D_61_ = 13.2	D_90_ = 26.6	7100	2850	Chemical effect
Poppe et al. (2005) [5]	(0–67%) LS + (33–100%) PC	–	D_50_ ≈ 10	D_50_ ≈1 7 (CEM I 42.5R); D_50_ ≈ 18 (CEM I 52.5); D_50_ ≈ 10 (CEM I 52.5 HSR LA)	5260	2810 (CEM Ⅰ 42.5R); 2860 (CEM I 52.5); 4180 (CEM I 52.5 HSR LA)	Nucleation effect, Chemical effect
Ye et al. (2007) [9]	(33–43%) LS + (57–67%) PC	–		–	5260	4200 (CEM I 52.5)	Nucleation effect
Lothenbach et al. (2008) [49]	PC4: (4%) LS + (96%) PC	–	Mean particle size: 4	–		4130 (PC); 4290 (PC4)	Chemical effect
Weerdt et al. (2011) [32]	(0–5%) LS + (0–35%) FA + (65–100%) PC	–	D_50_ = 4	D_50_ = 11	8100	4500	Chemical effect
Bentz et al. (2012) [50]	–	(0–10%) LS + (30–40%) FA + (55–100%) PC	D_50_ (median particle size of LS) = 4.4, 16.4; Nano-LS (nm): 50–120	D_50_ (median particle size) ≈ 20	–	4760	Nucleation effect; Chemical effect
Vance et al. (2013) [47]	–	(0–40%) LS + (0–10%) FA/MK + (50–100%) PC	D_50_ (median particle size) = 0.7, 3, 15	D_50_ ≈ 10	–	–	Nucleation (0.7 and 3 μm LS); Chemical effect
Zajac et al. (2014) [51]	Laboratory cement containing 15% of LS; Commercial cement containing 1%, 3%, 9% of LS, respectively	–	D_50_ = 8 (LS in laboratory cement)	–	7000 (LS in laboratory cement)	–	Nucleation effect; Chemical effect
Thongsanitgarn et al. (2014) [35]	(0–30%) LS + (0–30%) FA + (70–100%) PC; (0–15%) LS + (85–100%) FA	–	Maximum particle size: 5, 20	–	–	–	Nucleation effect, chemical effect (5 μm); Dilution effect (20 μm)
Bentz et al. (2015) [26]	(0–10%) LS + (0–20%) FA + (75–100%) PC	–	D_50_ = 1.58 (Fine LS); D_50_ = 15.7 (Coarse LS); D_50_ = 7.11 (Mmarblewhite); D_50_ = 3.09 (Sturcal F); D_50_ = 1.59 (HT Sturcal F)	D_50_ =10.6 (Type III cement); D_50_ = 9.85 (White cement); D_50_ = 11.9 (Type I/II cement)	–	4810 (Type III cement); 3970 (White cement); 3730 (Type I/II cement)	Nucleation effect (fine LS and calcite LS); Chemical effect (fine LS); Dilution effect (fine and coarse LS)
Schöler et al. (2015) [29]	(0–20%) LS + (0–30%) FA + (20–30%) BFS + 50% PC	–	D_50_ = 16	D_50_ = 11	4650	5180	Chemical effect

Notes: LS, PC, MK, FA, BFS represent limestone, Portland cement, metakaolin, fly ash and blast furnace slag, respectively; D_50_, D_61_, D_90_ represent the particle sizes of limestone powder when fraction passing are 50%, 61% and 90%, respectively; CEM and HSR LA represent different cement types.
